# Long-term outcomes of intravenous fibrinolysis in central retinal artery occlusion

**DOI:** 10.1038/s41598-023-47987-9

**Published:** 2023-11-22

**Authors:** Pavel Kozner, Libor Eichenmann, Marie Ceska Burdova, Marketa Pavlikova, Martin Hlozanek, Dagmar Dotrelova

**Affiliations:** 1 Department of Ophthalmology, Second Faculty of Medicine Charles University and Motol University Hospital in Prague, V Uvalu 84, 150 06 Prague, Czech Republic; 2 Department of Probability and Mathematical Statistics, Faculty of Mathematics and Physics, Charles University in Prague, Prague, Czech Republic

**Keywords:** Retinal diseases, Vision disorders, Retina, Atherosclerosis, Carotid artery disease, Thromboembolism, Ocular ischemic syndrome

## Abstract

Central retinal artery occlusion (CRAO) is an ophthalmologic emergency that can lead to irreversible loss of vision. Intravenous thrombolysis (IVT) has been used experimentally for its treatment. Our study aimed to evaluate the effect of emergency IVT on CRAO and its impact on visual acuity outcomes. We conducted a retrospective observational study of patients with CRAO. A total of 46 patients with CRAO were analysed; 16 patients received IVT treatment (IVT group) while 30 did not (no-IVT group). Seven patients from the IVT group received IVT early, within 4.5 hours (h) after the onset of symptoms (early-IVT), and 9 patients received it beyond this timeframe (late-IVT). The median time-to-hospital was 8.5 h: 3 h for the IVT group and 24 h for the no-IVT group. The median time-to-treatment was 5 h. The median outcome of visual acuity was 0.05 in the early-IVT, 0.025 in the late-IVT, and 0.01 in the no-IVT group. Among patients who received IVT early, 86% exhibited significant visual improvement. This improvement was four-fold greater compared to all other groups (p = 0.040), including the late-IVT (p = 0.011) and no-IVT groups (p = 0.023). No complications of the treatment were reported. Our study confirms that the administration of IVT treatment for CRAO within the 4.5-h time window is both safe and effective.

## Introduction

Central retinal artery occlusion (CRAO) represents a severe ophthalmic emergency that can lead to permanent vision loss in most patients^1,2^. The major cause of CRAO is thromboembolism; with fibrin-platelet emboli and thrombi being the most common culprits, followed by cholesterol-containing emboli or calcific detritus^3,4^. Inflammatory vessel disease is the least frequent cause, affecting only 5% of patients, and can be effectively treated with steroids^5^. Unlike inflammatory CRAO, treatment guidelines for non-inflammatory CRAO are yet to be established.

The classic conservative standard treatment (CST) for CRAO, which aims to reduce intraocular pressure and facilitate ocular perfusion, has not been proven superior over no treatment (NT)^6,7^. Therefore, medical centers have attempted alternative experimental treatment options involving fibrinolytics.

Several studies indicate that the use of local intra-arterial fibrinolysis (LIF) with recombinant tissue plasminogen activator (rtPA) presents many challenges. This treatment modality is less effective in treating CRAO compared to acute ischemic stroke (AIS) and is associated with a high incidence of complications^2^.

Therefore, intravenous thrombolysis (IVT) with rtPA has been prioritized. IVT is considered a safer and more feasible option, but it is also less effective than LIF^8,9^. Unlike LIF, which should be administered within 6 h post-AIS, IVT requires even more prompt administration—within a maximum of 4.5 h^8,10^.

To date, only a few observational clinical studies and limited meta-analyses have been published on IVT treatment in CRAO^6,7,11–13^.

The prevailing consensus lacks strong support for incorporating IVT into CRAO treatment guidelines, and it is still considered an experimental treatment. Our paper presents 15 years of experience with IVT in CRAO to discuss feasibility, risks, and effectiveness of the treatment.

Our study aimed to assess the effect of emergency IVT on CRAO and its influence on visual acuity improvement. The primary objective was to compare visual acuity outcomes between patients who underwent IVT and those who did not. Our secondary objective was to identify factors that contribute to the successful restoration of visual function, including time-to-treatment and time-to-hospital, the underlying causes of CRAO, the frequency of ocular ischemic complications, and the use of additional anticoagulant or antithrombotic treatments.

## Method

A retrospective observational cohort study was conducted to examine the diagnosis of CRAO and its treatment at our hospital. The medical records of patients were meticulously searched for this purpose. The study enrolled individuals who were 18 years of age or older and had been diagnosed with CRAO during their admission to Motol University Hospital in Prague between 1st January 2006 and 31st December 2021. The exclusion criteria included underlying inflammatory vessel disease and incomplete records for statistical analyses.

The following data were collected: age, gender, race, affected side, the time from the onset of symptoms to hospital admission (time-to-hospital), the time from the onset of symptoms to the treatment initiation (time-to-treatment), blood and imaging results, additional anticoagulant or antithrombotic treatment (ACTx), best-corrected visual acuity on initial presentation with CRAO (BCVA1), best-corrected visual acuity on follow-up (BCVA2), complications of IVT treatment, ocular ischemic complications of CRAO, the principal cause of CRAO, and survival after CRAO.

Two main arms of the cohort were established. The first arm comprised patients with CRAO who were treated with IVT (IVT group), while the second arm consisted of patients with CRAO who did not receive IVT (no-IVT group). The IVT group was further subdivided into two subgroups based on the timing of IVT administration: the early-IVT subgroup included patients who received IVT within 4.5 h after symptom onset, while the late-IVT subgroup included patients who received IVT between 4.5 and 12 h after symptom onset.

The following pathway was shared for all patients with CRAO at our hospital: Confirmation of CRAO by an on-call ophthalmologist following a routine ophthalmology review. Diagnosis of CRAO was based on clinical symptoms and objective findings observed during emergency ophthalmology assessments, with a particular focus on identifying ischemic retinal changes on fundoscopy in association with severely reduced vision; baseline blood pressure; laboratory tests—blood count, coagulation, erythrocyte sedimentation rate (ESR) and biochemical tests to exclude giant cell arteritis (GCA); pre-treatment computed tomography (CT) or CT angiogram (CTa) of the brain to exclude cerebral infarction, intracranial hemorrhage, or mass lesion.

Informed consent for IVT was obtained from all subjects or their legal guardians prior to the treatment. Alteplase was administered by intravenous infusion (0.9 mg/kg) by a consultant neurologist in the Stroke unit as per the standard protocol in accordance with relevant guidelines for AIS. A follow-up CT scan of the brain has been obtained 24 h after IVT for safety reasons. Exclusion criteria for IVT included a recent history of AIS or systemic hemorrhage, ischemia or hemorrhage on CT scan, factors known to increase the risk of hemorrhage, and a calculated time to treatment of more than 12 h.

Baseline visual acuity was tested using the best-corrected visual acuity (BCVA) method with the Snellen chart on initial presentation with CRAO (BCVA1). The Snellen chart is our emergency room standard. Post-treatment best-corrected visual acuity (BCVA2) was measured either by the Snellen or decimal charts at follow-up in our clinics. The best readings obtained at the follow-up within the one month after CRAO were recorded.

To unify the visual acuity readings, all Snellen fractions and low vision outcomes were converted to decimal equivalents. To quantify the off-chart vision, we used the conversion to decimal values by Holladay; 0.01 for “counting fingers” (CF) and 0.001 for “hand movement” (HM)^14^. To preserve the extremely low vision outcomes for analysis, Holladay’s conversion was expanded with a value 0.0001 assigned for “light perception” (LP) in the extended conversion (Table 1).

**Table 1:** Conversion chart of vision categories.

Actual vision	Holladay’s conversion*	Extended conversion	Category of vision
1.0 (standard vision)	1	1	0
0.1 (on-chart)	0.1	0.1	− 1
Counting fingers (CF)	0.01	0.01	− 2
Hand movements (HM)	0.001	0.001	− 3
Light perception (LP)	0	0.0001	− 4
No light perception (NPL)	0	0.000001	− 6

Category of vision corresponds to log10 transformation of the extended conversion.

*Holladay^14^.

Consequently, all decimal readings were converted to logarithmic values for statistical analysis. In principle, the visual acuity of patients with CRAO tends to have abnormal distribution towards the low values, so a parametric approach was considered preferable to detect the trends in vision contrary to previous studies. Therefore, six categories of vision (category) corresponding to BCVA were established: standard vision (1.0 = category 0), on-chart vision (1e−01 = category − 1), CF (1e−02 = category − 2), HM (1e−03 = category − 3), and LP (1e−04 = category − 4). Intermediate values were rounded to the nearest category. A particular category for consideration was no perception of light (NPL). As a rule, it is impossible to create a zero logarithmic value. To preserve NPL for statistical analyses, a value 1e−06 and category − 6 were added to the extended conversion for NPL instead of zero (Table 1).

Vision improvement or deterioration was determined as a change in the vision category between the BCVA1 and BCVA2. Improvement was defined as an upward shift of at least one category and deterioration as a downward shift of at least one category. The change in vision categories was tested in relation to the reception of IVT and compared in the IVT group, no-IVT group, early-IVT and late-IVT subgroups, respectively.

Underlying cause of CRAO was divided into four groups: carotid stenosis (advanced carotid stenosis), atherosclerosis (atherothrombotic), thrombus (thromboembolic) and unknown. The cause “carotid stenosis” was set in patients with significant stenosis of the ipsilateral carotid of 80% or more. “Thrombus” was flagged in patients with coagulopathy or right-to-left heart shunt on echocardiography. “Atherosclerosis” was marked in patients with atherosclerosis on duplex sonography, carotid stenosis under 80%, history of myocardial infarction, AIS or peripheral arterial disease. The underlying cause of CRAO was tested against the visual outcomes in the IVT and non-IVT groups.

Ocular ischemic complications of CRAO were recorded within a period of six months after the episode of CRAO: retinal, disc or iris new vessels (as well as the presence of neovascular glaucoma, history of laser coagulation or anti-VEGF therapy). The frequency of the ocular complications was tested in both, the IVT and no-IVT groups.

Besides the IVT, additional ACTx was also studied. Patients were divided into 3 main subgroups according to the type of ACTx: low molecular weight heparin (LMWH), dual anti-platelet therapy with acetylsalicylic acid and clopidogrel (DAPT), and other monotherapy or combined therapy (others). ACTx with LMWH, DAPT and others was tested against the patients’ visual outcomes in IVT and no-IVT groups.

Life status was verified throughout the healthcare system registry as of 31st December 2021 to determine whether the patient was still alive or deceased.

All methods used in this study were carried out in accordance with relevant guidelines and regulations. Data were summarized as absolute and relative frequencies for categorical variables and as means with standard deviation (SD) or medians with interquartile range (IQR) for continuous variables. Comparisons of various characteristics groups (such as IVT vs no-IVT or male vs female) were carried out using Fisher’s exact test for categorical and t-test or Wilcoxon’s two-sample test for continuous variables. Data on vision at admission or vision changes were analyzed using either categorical data or a continuous data approach. In ordered categories, comparisons between IVT vs no-IVT groups were performed using a linear-by-linear test. We used log-transformed vision data for the continuous data approach and compared them using t-tests and linear regression models, with various patient characteristics, including time-to-hospital and time-to-treatment as independent variables. All analyses were carried out with I statistical language and environment R, version 4.1.0.

### Ethics approval

All procedures performed in study involving human participants were in accordance with the ethical standards of Good Clinical Practice of Institutional Review Board and all methods were approved by Ethics Committee of the University Hospital Motol and 2nd Faculty of Medicine, Charles University in Prague, and with the 1964 Helsinki declaration and its later amendments or comparable ethical standards.

### Consent for treatment

Informed consent was obtained from all individual participants included in the study.

## Results

Forty-eight adult patients were admitted to our hospital with diagnosis of CRAO between January 2006 and the end of December 2021. Two patients were excluded from the statistical analysis: the first patient due to diagnosis of GCA and the second due to incomplete medical records. Patients’ characteristics are presented in Table 2.

**Table 2:** Patients’ characteristics.

Patients’ characteristics: categorical variables	IVT group (N = 16)		No-IVT group (N = 30)		Total (N = 46)		Comparison between IVT and no-IVT group p-value*
	n	%	n	%	n	%	
Gender—M/F	13/3	81.2/18.8	18/12	60.0/40.0	31/15	67.4/32.6	0.195
Affected side—right/left	9/7	56.2/43.8	19/11	63.3/36.7	28/18	60.9/39.1	0.754
CRAO cause							0.163
Atheriosclerosis	5	31.2	15	50	20	43.5	
Thrombus	3	18.8	6	20	9	19.6	
Carotid stenosis	8	50.0	6	20	14	30.4	
Other	0	0.0	3	10	3	6.5	
Time to treat window							
Under 3 h	4	25.0					
3–4.5 h	3	18.8					
4.5–6 h	3	18.8					
6–12 h	6	37.5					

Patients’ characteristics: continuous variables	Mean (SD)	Range	Mean (SD)	Range	Mean (SD)	Range	p-value^#^
Age (years)	67.1 (13.0)	29–84	69.4 (9.4)	50–86	68.6 (10.7)	28–86	0.486
BCVA1 category	− 2.6 (1.2)	− 4 to 0	− 3 (1.1)	− 6 to − 1	− 2.9 (1.1)	− 6 to 0	0.279

Patients’ characteristics: continuous variables	Median (IQR)	Range	Median (IQR)	Range	Median (IQR)	Range	p-value^§^
BCVA1	0.0010 (0.0092)	0.0001–0.9	0.0010 (0.0097)	0–0.1	0.0010 (0.0097)	0–0.9	0.546
Time-to-hospital (h)	3.0 (1.5–6.1)	1–9	24 (8–90)	1–4032	8.5 (3.2–48)	1–4032	< 0.0001
Time-to-treatment (h)	5.0 (3.8–7.9)	2–12	–	–	–	–	–

*IVT* intravenous thrombolysis, *CT* computed tomography, *BCVA1* initial best corrected visual acuity on first presentation, *M* male, *F* female, *IQR* interquartile range, *range* minimum to maximum.

*p-value of Fisher’s exact test.

^#^p-value of t-test.

^§^p-value of Wilcoxons’s two-sample test.

We analyzed 46 patients, 16 patients (34.8%) received IVT (IVT group), 30 patients (65.2%) not (no-IVT group). The median time-to-hospital from the onset of symptoms was 8.5 h (IQR 3.2–48.0), 3 h in the IVT (IQR 1.5–6.1 h) and 24 h in the no-IVT group (IQR 8–90 h), respectively. The IVT administration was not related to any variable other than time-to-hospital and time-to-treatment.

The median time-to-treatment was 5 h (IQR 3.8–7.9). In the IVT group, seven patients (43.75%) received IVT within 4.5 h after onset of symptoms (four patients within 3 h, three patients between 3 and 4.5 h) and were marked as early-IVT; nine patients (56.25%) received IVT later (three patients between 4.5 and 6 h and six patients later than 6 h) and were designated as late-IVT.

The median BCVA1 was 0.001 (HM) in all patients, the no-IVT group, IVT group and early-IVT subgroup, respectively; 0.01 (CF) in the late-IVT subgroup. The median BCVA2 was 0.01 (CF) in all patients and in the no-IVT group, 0.039 in the IVT group, 0.05 (20/400, 6/120) in the early-IVT subgroup, and 0.025 in the late-IVT subgroup, respectively (Table 3).

**Table 3:** Change in vision by groups.

	BCVA1	BCVA2	BCVA1 category	BCVA2 category	BCVA category change	t-test p-value	F-test p-value
	Median (IQR)	Median (IQR)	Mean (SD)	Mean (SD)	Mean (SD)		
All (N = 46)	0.0010 (0.0097)	0.010 (0.099)	− 2.9 (1.1)	− 2.1 (1.4)	0.8 (1.4)	0.147*	0.040^#^
No-IVT (N = 30)	0.0010 (0.0097)	0.010 (0.028)	− 3.0 (1.1)	− 2.5 (1.5)	0.5 (1.5)		
IVT (N = 16)	0.0010 (0.0092)	0.039 (0.103)	− 2.7 (1.2)	− 1.5 (1.1)	1.2 (1.2)		
Early-IVT (N = 7)	0.0010 (0.0009)	0.050 (0.781)	− 3.0 (1.4)	− 1.0 (0.9)	2.0 (1.3)		
Late-IVT (N = 9)	0.010 (0.001)	0.025 (0.090)	− 2.4 (1.0)	− 1.8 (1.1)	0.5 (0.7)		

*IVT* intravenous thrombolysis, *BCVA1* initial best corrected visual acuity on first presentation, *BCVA2* final best corrected visual acuity on follow-up, *IQR* interquartile range, *SD* standard deviation.

*Comparison of IVT vs. no-IVT group by t-test.

^#^Comparison of no-IVT vs. early-IVT vs. late-IVT groups by ANOVA F-test.

Other pairwise comparisons: early-IVT vs. late IVT p = 0.014 (t-test), early-IVT vs. no-IVT p = 0.023 (t-test).

No statistically significant difference was determined in the distribution of BCVA1 and BCVA2 across the groups (p = 0.279, F-test ANOVA), nor in the change of vision categories between the IVT and no-IVT group (t-test 0.147).

**Figure 1:**
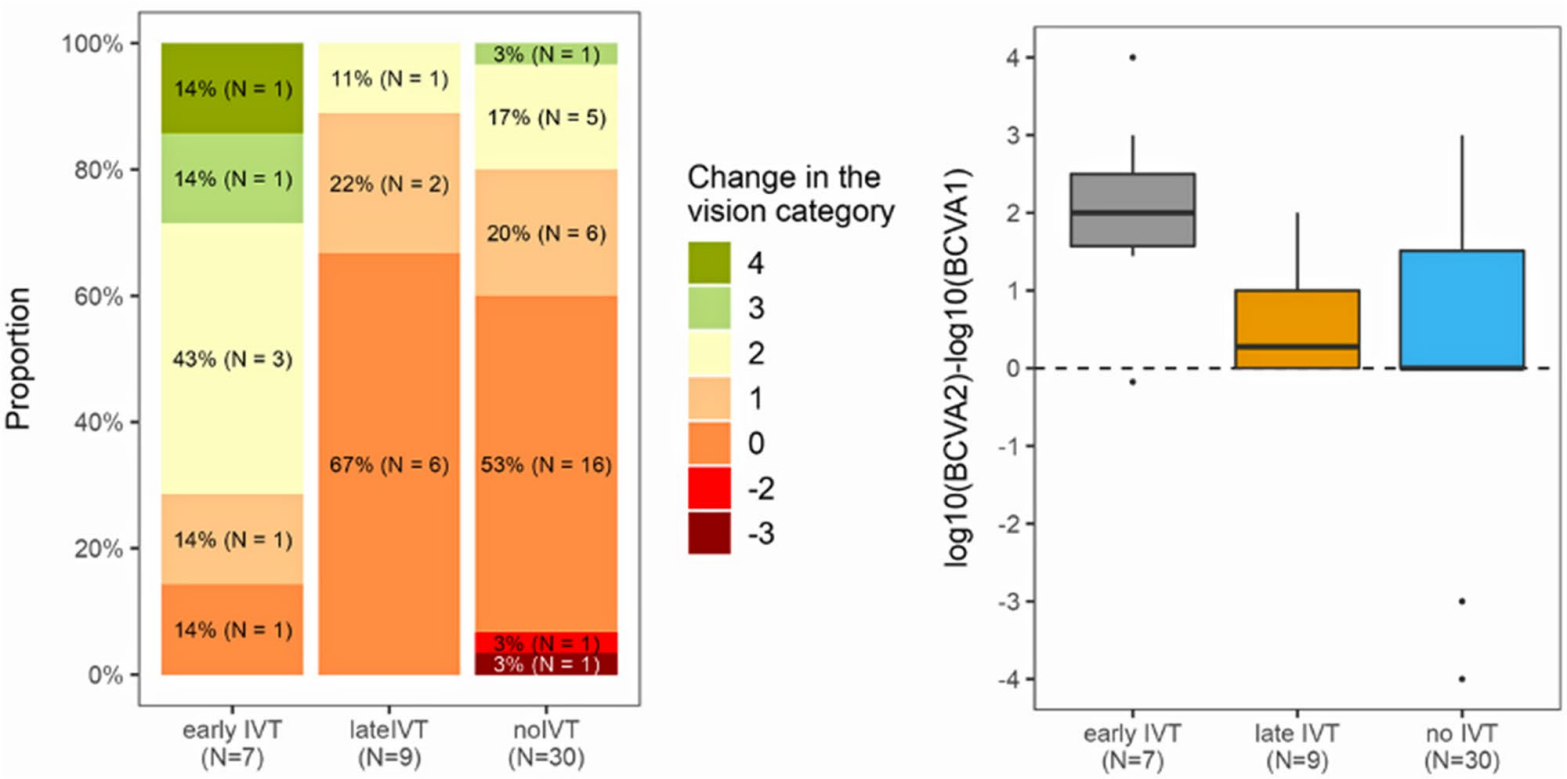
Change of vision in dependence in relation to treatment.

However, a statistically significant difference was determined in the time-to-treatment analysis, with the early-IVT subgroup showing a significant improvement of vision compared to both, the late-IVT subgroup and the no-IVT group (p = 0.040, F-test ANOVA). Six out of seven (86%) patients in the early-IVT subgroup exhibited significant vision improvement, with an average improvement by two vision categories. This improvement was significantly greater than that observed in the late-IVT subgroup (0.5 category, p = 0.011) or the no-IVT group (0.5 category, p = 0.023, pairwise t-test comparisons) (Table 3, Fig. 1).

Two out of 16 treated patients (12.5%) experienced co-morbidities. One patient suffered a small self-limiting occipital brain infarction, and the other had an acute renal failure, both were treated successfully during the admission. No patient deceased in consequence of IVT in our observational cohort.

No statistically significant difference was determined in the distribution of major causes of CRAO between the IVT and no-IVT groups (p = 0.521). According to our analysis, visual improvement was not associated with any principal cause of CRAO in either group. (p = 0.94, F test ANOVA).

Ocular ischemic complications of CRAO were identified in six patients in the IVT group (37.5%) and eight patients in the no-IVT group (26.7%) at six months. There was no statistical difference in the frequency of these complications between the IVT and no-IVT groups (p = 0.512).

**Figure 2:**
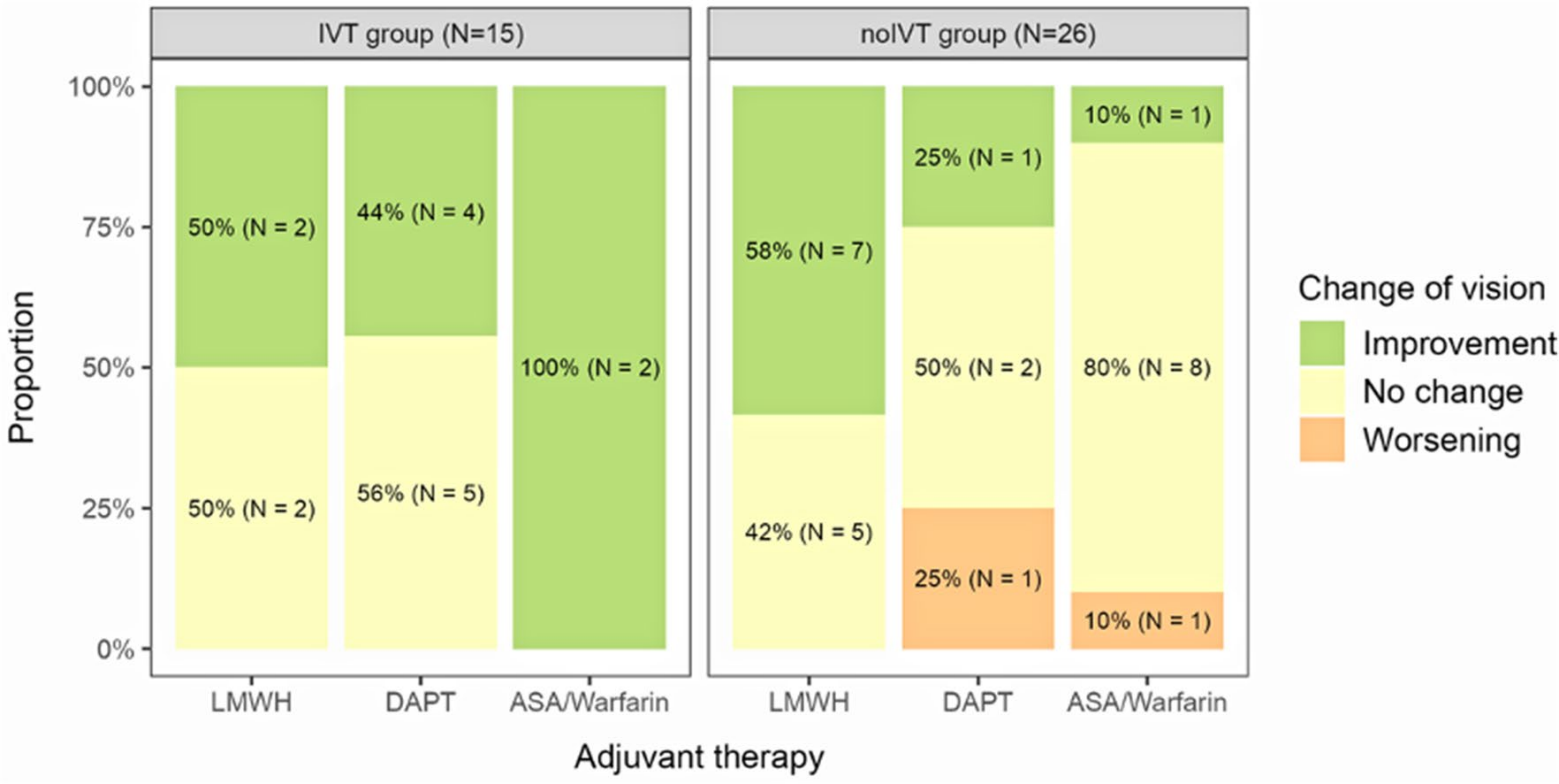
Effect of additional therapy on visual improvement.

Additional ACTx did not affect the visual outcomes in our patients in a statistically significant manner (p = 0.333). However, the intervention with LMWH in the no-IVT group was associated with a better visual prognosis compared to other types of ACTx or no treatment (median 1.26 (IQR 2) category vs 0 (IQR 0), p = 0.032, see also Fig. 2). No patients received intraocular lowering therapy for CRAO in examined group.

The visual acuity on follow-up was not affected by age (p = 0.349). The survival rate did not differ significantly between the IVT and no-IVT groups (p = 0.27).

## Discussion

According to previous studies, 80% of patients with CRAO have severely reduced visual acuity upon their initial visit, falling into the low vision range. Nearly 90% of them are left with such a low vision permanently^1,2^. Our study is consistent with these findings, with median BCVA of HM upon admission, and median BCVA at the level of CF on follow-up.

Based on the available meta-analyses, spontaneous visual recovery in CRAO is 17.7% without any treatment (NT), 7.4% with conservative treatment (CST), and 31.8% with time unlimited thrombolysis^7^. Accepting that NT is as ineffective as CST there are few options other than fibrinolysis for CRAO, LIF or IVT^15,16^.

Administering LIF via microcatheter to the ophthalmic artery is a highly complex procedure that contradicts the urgency to initiate recanalization treatment at the nearest hospital as quickly as possible. The safety of LIF is the second concern; studies suggest that up to 30% of CRAO patients may have coincidental cerebral ischemia on MRI, which is a significant risk factor for intracerebral haemorrhage associated with LIF^17^. Both issues have contributed to the low clinical acceptance of LIF as a primary treatment for CRAO. In contrast, IVT appears to be a more practical option, as it significantly reduces complications, and most emergency personnel can administer it. The only limitation of IVT is the narrow time window for treatment^8^.

**Figure 3:**
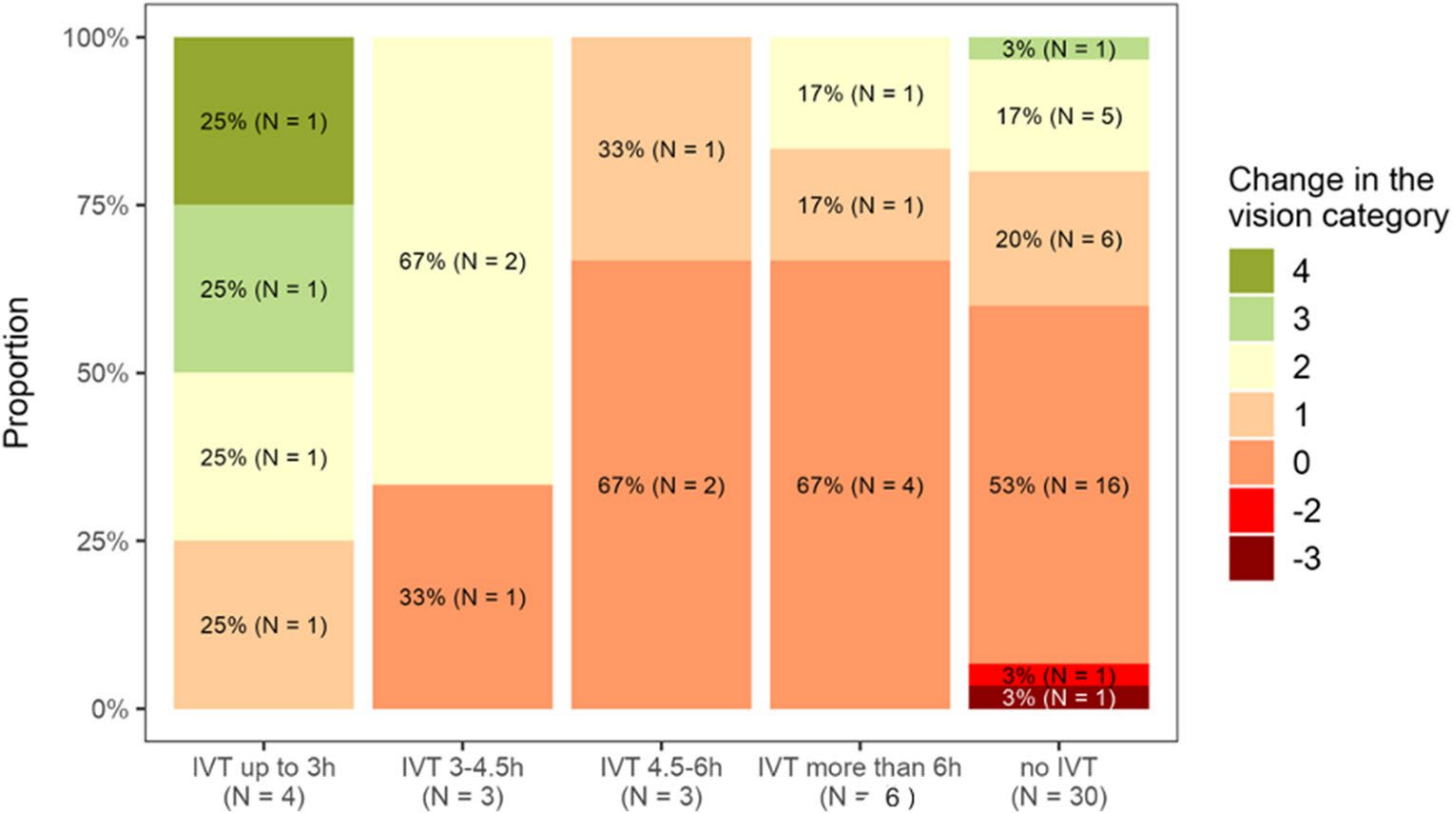
Trends in visual recovery in relation to treatment.

Our study has confirmed that administering IVT within 4.5 h of the onset of CRAO symptoms is crucial for successful treatment, as also shown in Fig. 3. However, previous studies conducted by neurologists lacked detailed information on mean visual acuity gains, visual recovery calculation methodology, and appropriate categorization of visual acuity toward low vision values, resulting in recovery rates of only 44–50%. Moreover, the definition of treatment success varied across these studies and did not meet the standards required for consideration of ophthalmology protocols^6,7,16^.

In contrast, our study has made significant progress in this area. By accurately categorizing low vision values, we have found that up to 86% of patients who received IVT within 4.5 h experienced substantial visual improvement, on average by two vision categories of low vision scale. This improvement is four times better than the outcomes observed in patients treated later or left untreated, indicating a significant improvement of vision in most of the treated patients. From our perspective, success in treatment is determined by the individual's ability to read and their level of independence. On follow-up, our patients who received IVT within 4.5 h of symptom onset had a median best-corrected visual acuity of 0.05 decimal (20/400 or 6/120 Snellen). This is the last level of severe low vision that is on the chart, allowing patients to read slowly with reading aids and high-power magnifiers and to preserve their independence^18^.

Our study did not find any indication that either age or the specific underlying cause of CRAO resulted in better outcomes from IVT treatment in terms of visual recovery. Furthermore, there was no significant difference in the incidence of ocular neovascular complications between the IVT and no-IVT groups at the six-month follow-ups. However, this finding is inconclusive because ocular ischemic complications of CRAO may manifest with a delay. Although we anticipated a higher total number of neovascular complications in CRAO, the distribution between the IVT-treated and untreated groups in our study was even.

Additional anticoagulant or antithrombotic treatment has been widely used in the AIS protocols. Most of our IVT-treated patients received ACTx following the IVT at a prophylactic dose**,** while most of IVT-untreated patients received ACTx at a therapeutic dose. We could not determine the overall benefit of any ACTx on visual recovery. Interestingly, LMWH intervention was associated with a better visual prognosis in IVT-untreated patients, compared with other types of ACTx or no ACTx. We hypothesize that the complex patients who are not suitable for IVT, or patients who are beyond the IVT treatment window**,** receive LMWH treatment at a therapeutic dose instead. LMWH at a therapeutic dose may affect thrombus dissolution similarly to IVT. Future research is subject to additional investigation of this finding.

Central retinal artery occlusion is a rare condition, leading to most research featuring small sample sizes. Prospective studies on CRAO pose even greater challenges due to low incidence and the condition's typical occurrence during non-standard hours. Nevertheless, we are pleased to present one of the most extensive patient populations treated with IVT for CRAO in a single medical centre and share our protocol with favourable results.

In conclusion, we have confirmed that the time-to-treatment is the sole independent factor that impacts the success of IVT treatment in terms of visual recovery in CRAO. Defining treatment success in terms of independence and ability to read, our study has demonstrated that it can be achieved in the majority (86%) of early treated patients. This finding strongly encourages early administration of IVT treatment for all patients with CRAO who attend to hospital in time. With respect to treatment protocols and the 4.5-h time frame, IVT appears to be safe and efficient treatment for CRAO**.** Therefore, guidelines and hospital treatment protocols should be re-evaluated.

## Data Availability

The datasets analysed during the current study are available from the corresponding author on reasonable request.
